# Surface-Enhanced Raman Spectroscopy Detection of Per- and Polyfluoroalkyl Substances in Aqueous Film-Forming Foams

**DOI:** 10.1021/acs.est.5c10183

**Published:** 2025-12-21

**Authors:** Chuntao Wang, Kushal Biswas, Sangmin Jeong, Anila Bello, Dhimiter Bello, Michael B. Ross

**Affiliations:** † Department of Chemistry, 14710 University of Massachusetts Lowell, Lowell, Massachusetts 01854, United States; ‡ Department of Biomedical and Nutritional Science, 14710 University of Massachusetts Lowell, Lowell, Massachusetts 01854, United States; § Department of Public Health, 14710 University of Massachusetts Lowell, Lowell, Massachusetts 01854, United States

**Keywords:** per- and polyfluoroalkyl substances (PFAS), aqueous film-forming foams (AFFFs), surface-enhanced Raman spectroscopy (SERS), gold nanoparticles

## Abstract

Identifying the presence and identity of per- and polyfluoroalkyl substances (PFAS) in complex mixtures is critical for water treatment, hazardous waste cleanup, and the identification of workplace hazards. The cost and scale of conventional methods for PFAS detection often make rapid and portable detection challenging. Here, we use concave cubic gold nanoparticles for surface-enhanced Raman spectroscopy (SERS) to detect PFAS in parts per million concentrations, differentiating the 6 PFAS—PFHpA, PFNA, PFDA, PFOA, PFHxS, and PFOS—regulated in the Commonwealth of Massachusetts by the Department of Environmental Protection (MassDEP). Calculated Raman spectra, solid-state Raman spectra, and ^19^F NMR are used to further understand the physicochemical properties of these 6 PFAS. Quantitative analysis of PFOA and PFOS can be achieved from 0.1 to 10 ppm, while PFAS can be differentiated from three common fluorinated pharmaceuticals, and perfluoroalkyl carboxylic acids (PFCA) can be differentiated from C7 to C10 based on the length of the perfluoroalkyl backbone. Finally, we highlight that SERS can be used to identify PFAS in real-world aqueous film-forming foams (AFFFs), as confirmed separately by mass spectrometry. These results advance our ability to detect and analyze PFAS in real-world samples relevant to environmental monitoring and analysis.

## Graphical abstract

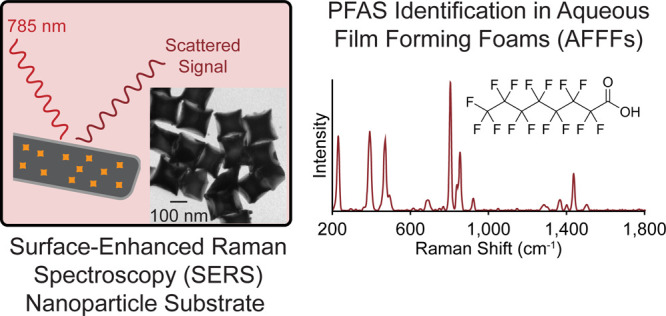

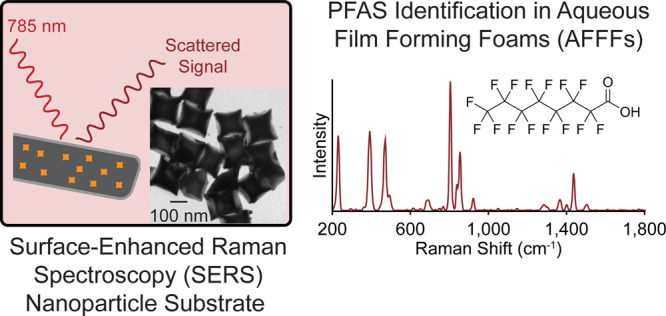

## Introduction

Per- and polyfluoroalkyl substances (PFAS) make up a group of synthetic chemicals that are widely used in industrial and consumer products. They are persistent and recalcitrant pollutants that resist natural degradation processes, leading to long-term environmental contamination and a variety of human health effects.^1–3^ Detecting and quantifying PFAS is highly challenging due to their regulation at extremely low concentrations (e.g., ppt levels), their extensive chemical structural diversity, and their presence in complex multicomponent mixtures.^4^ The identification of PFAS in foams and nontraditional water sources is challenged by the complex chemical composition of these matrices, which can interfere with detection methods and reduce sensitivity. Factors such as surfactants, organic compounds, and varying pH levels complicate the extraction process, while the structural diversity of PFAS compounds necessitates highly sensitive and selective techniques for accurate identification under diverse environmental conditions. Previous research used a combination of Raman and SER spectra with machine learning techniques, achieving highly sensitive detection and quantification of PFAS.^5^ Traditional methods, such as liquid chromatography-tandem mass spectrometry (LC-MS/MS), have long been used for PFAS analysis because they offer high sensitivity, specificity, and accuracy, and they continue to be the gold standard in PFAS quantification. However, their complexity, high cost, and need for in-lab instrumentation and processing limit their applicability outside the lab. Furthermore, even the methods with the best analyte coverage (50–70 analytes per run) miss a substantial number of PFAS species from the thousands of PFAS structures in commercial use. Thus, rapid detection of PFAS in environmental samples and in the workplace (e.g., fire stations) remains a highly desirable capability and currently an unmet challenge.

One common use of PFAS is in firefighting aqueous film-forming foams (AFFFs), which contain a combination of fluorochemical surfactants and other additives. AFFF typically contains high concentrations of PFAS due to their formulation for rapid firefighting applications, often leading to significant localized contamination. Timely detection and precise quantification of PFAS in environments affected by AFFF deployment are essential to the environment and public health risks due to the high mobility of these compounds in soil and aquatic systems. The complexity of the AFFF matrix makes it challenging for rapid analysis. Raman spectroscopy, in principle, offers advantages for samples such as these due to its speed and nondestructive analysis, but challenges persist in achieving the desired sensitivity and selectivity requirements for low-level PFAS detection.^1,6–8^

Surface-enhanced Raman spectroscopy (SERS) is a powerful analytical approach that can be used to detect trace amounts of chemicals with the advantage of high sensitivity and selectivity, rapid analysis, and potential for portability.^9^ SERS utilizes plasmonic nanoparticles, which can be altered by changing size and morphology, that enhance electromagnetic fields near the nanoparticle surface to increase the Raman scattering cross-section of molecules, resulting in a stronger Raman scattering signal and higher detection sensitivity.^10^ Previous work showed that extracted fluorosurfactants could be detected using dye-assisted SERS on Ag nanoparticles; however, this strategy adds complexity due to the need for a partner SERS dye molecule.^11^ Direct detection of PFAS using SERS remains challenging due to their chemical complexity and relative lack of chemical handles to facilitate specific adsorption.^7^ With specifically designed plasmonic nanoparticle substrates, it contributes to an increase in sensitivity of SERS sensing and differentiation of PFAS in water, achieving a wide range of detection from ppb to ppm.^12^ Herein, we used Au concave cubic nanoparticles as a substrate to enhance the Raman signal and improve the PFAS detection sensitivity.^11^ Such a platform for rapid and portable analysis of PFAS in complex matrices would be transformative for managing PFAS exposure, understanding their environmental distribution, and limiting their accumulation.

## Results and Discussion

Au concave cubic nanoparticles have sharp corners and edges that can act as hotspots for SERS (Figure 1a,b).^8,13^ Au concave cubic nanoparticles were synthesized as reported previously and detailed in the Methods.^14^ Scanning electron microscopy (SEM) and transmission electron microscopy (TEM) (Figure 1a,b) revealed the successful synthesis of approximately 80 nm concave cubes. UV–visible spectroscopy indicated that the localized surface plasmon resonance (LSPR) of the Au concave cubic nanoparticles is centered near 785 nm, aligning with the excitation wavelength for SERS studies. This specific design and wavelength optimization contribute to the enhanced sensitivity and performance of the SERS platform.^8,15–18^

**Figure 1:**
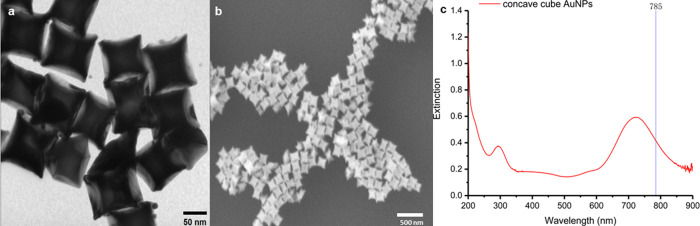
Structure and optical properties of Au concave cubic nanoparticles. (a) TEM and (b) SEM micrographs of Au concave cubic nanoparticles. (c) UV–visible spectrum of Au concave cubic nanoparticles.

We identified six specific PFAS standards for analysis according to those regulated in public drinking water by the Massachusetts Department of Environmental Protection (MassDEP). These PFAS include: perfluoroheptanoic acid (PFHpA), perfluorooctanoic acid (PFOA), perfluorononanoic acid (PFNA), perfluorodecanoic acid (PFDA), perfluorohexanesulfonic acid (PFHxS), and perfluorooctanesulfonic acid (PFOS). The molecular structure of four of the PFAS is linear and consists of an n-C chain with a terminal carboxylate. Two of the PFAS, PFHxS, and PFOS, contain terminal sulfonate head groups (Figure 2a).^2^

**Figure 2:**
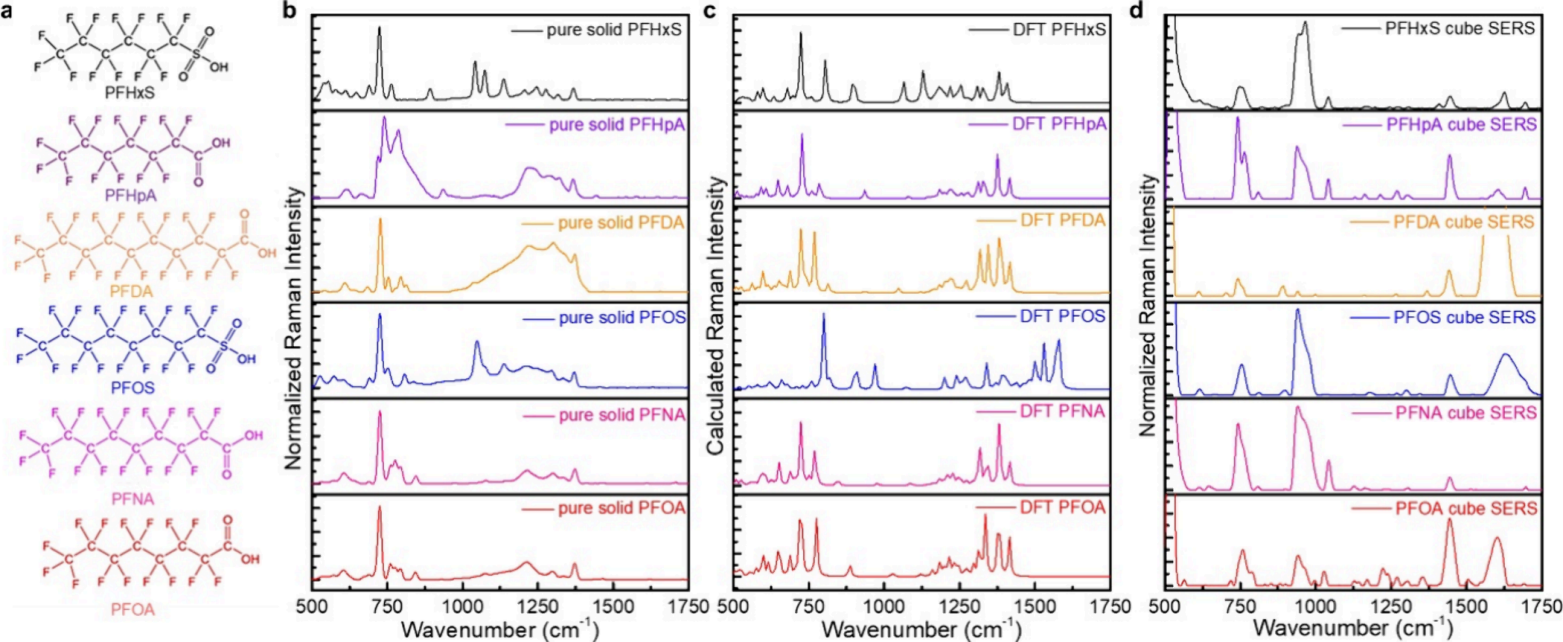
Chemical structure of MassDEP-regulated PFAS and comparison of solid, calculated, and surface-enhanced Raman spectra. (a) Chemical structures, (b) pure bulk Raman spectra, (c) DFT-calculated Raman spectra, and (d) SER spectra of the 6 PFAS. The SER spectra were measured for PFAS diluted to 10 ppm.

Raman spectra of solid-phase PFAS were collected using a 785 nm laser (Figure 2b). Theoretical Raman spectra were calculated using density functional theory (DFT) and are shown in Figure 2c; the vibrational modes were also tabulated as (Tables S1–S6). DFT is a quantum chemical computational method used to predict and interpret molecular structures, electron distributions, spectral information, and vibrational modes.^19,20^ DFT provides detailed theoretical vibrational frequencies and Raman scattering intensities analysis.^19,21^ It provides a comparison with the corresponding SER spectra, including peak positions and relative intensities.^22^ The calculated vibrational wavenumber shifts with respect to the molecule, and the calculated Raman intensities of the different PFAS complexes are compared with the experimental results.^21,23^ From previous research and the calculated results, the CF_2_ bond-stretching mode is located near 735 cm^–1^ and CF bond-stretching mode is located near 1350 cm^–1^.^24,25^ The C–C bond-stretching mode and twisting vibration mode are located between 1050 and 1150 cm^–1^, the SO_3_ stretching modes around 1045 and 1140 cm^–1^, and the COO^–^ stretching modes around 1400 and 1700 cm^–1^.^24,26^ Figure 2b,c shows a strong Raman peak intensity near 750 cm^–1^ and a relatively high-intensity peak at 1375 cm^–1^ for all six PFAS. Comparing PFOS and PFHxS with the rest of the four PFAS, there is SO_3_ in the molecular structure (Figure 2a), which appears as features at 1065 and 1125 cm^–1^.^24–26^

In a comparison of the Raman spectra obtained from DFT calculations with those from the solid-state PFAS powders, the peak positions and peak intensities are generally consistent. For example, when analyzing the CF and CF_2_ bond-stretching mode peaks, which are the most useful specific functional groups of PFAS for detection, the relative peak positions and intensities are consistent. The CF_2_ bond-stretching mode is near 720 cm^–1^ in DFT-calculated spectra and near 725 cm^–1^ in solid-state PFAS. The CF bond-stretching mode vibration peak is near 1370 cm^–1^ in DFT-calculated spectra and 1375 cm^–1^ in solid-state PFAS. One distinction is that the features of the PFAS functional groups are red-shifted by approximately 5–10 cm^–1^ in solid-state pure PFAS compared with DFT-calculated results.

Next, SERS of the same 6 PFAS was measured on concave cube substrates. These measurements were performed by first dissolving 0.01 g of PFAS in 10 mL of ultrapure water to prepare a 1000 ppm stock solution that was then diluted to 10 ppm. To prepare the nanoparticles as a SERS substrate, the nanoparticles were purified by centrifugation, washed with water, and drop-cast onto a silicon wafer surface. Five drops of the 10 ppm PFAS solution were then drop-cast and allowed to dry, after which SER spectra were acquired for each sample (Figure 2d). To eliminate the potential influence of PFAS contamination in ultrapure water, we prepared ultrapure water on the nanoparticle surface and obtained the SER spectrum (Figure S7).

To demonstrate the quantitative capability of SERS, we designed a calibration curve using PFOS and PFOA concentrations ranging from 0.1 to 10 ppm. The Raman intensity of the CF_2_ symmetric stretch near 750 cm^–1^ and the SO_3_ stretch near 1050 cm^–1^ were both used for quantification. As shown in Figures S9 and S10, the integrated peak intensities show a strong linear correlation with PFOS concentration (*R* ^2^ > 0.92) and PFOA concentration (*R* ^2^ > 0.88), indicating reliable semiquantitative detection within this range. These results confirm that SERS can be used not only for qualitative identification, but also for concentration-dependent quantification of PFAS under controlled conditions.

When compared with DFT-calculated PFAS Raman spectra and solid-state PFAS Raman spectra, SER spectra of six specific PFAS presented a strong peak within the range of 735–755 and 1350–1385 cm^–1^, which are the CF_2_ stretching mode and CF stretching modes, respectively. There are also two peaks near 950 and 1650 cm^–1^, which are due to residual adsorbed CTAC from the synthesis.^25,27–29^ The SER spectra show the C–F bond-stretching mode peaks red-shifted compared to the solid bulk and DFT-calculated ones. This is attributed to the Au plasmonic nanostructures, where strong dipoles may cause a red shift in the vibrations.^30–32^ Another plausible mechanism is the chemical effect that encompasses interactions between the molecules and plasmonic nanostructures, where the vibrations can be influenced by the chemical environment, adsorption geometry, electron density distribution, and electromagnetic enhancements on the SERS-active substrate.^9,33^

To better understand the impact of Au concave cubic nanoparticles on Raman signal intensity, we designed experiments using a sample with only Au concave cubic nanoparticles, a 10 ppm PFOS solution drop-cast on a clean Si wafer surface without Au nanoparticles, and drop-cast onto a Si wafer surface with Au concave cube nanoparticles (Figure S4). The spectra showed that when using only Au concave cubic nanoparticles, peaks near 950 and 1650 cm^–1^ were observed, corresponding to the surfactant CTAC. In contrast, PFOS measured without Au nanoparticles exhibited no detectable Raman signals from CF, CF_2_, or SO_3_ vibrational modes. At the same time, the detection limit of SERS with Au concave cubic nanoparticles was quantified (Figure S5). It is observed that there are no characteristic peaks for CF, CF_2_, or SO_3_ when the PFAS concentration is below 0.1 ppm. These characteristic peaks could still be observed at a concentration of 100 ppm, indicating that the method helps reduce the fluorescence of PFOS interference, which can interfere with the Raman signal.^15^

To further test the specificity of the SERS method, we analyzed three common fluorinated pharmaceutical compounds, atorvastatin (trade name Lipitor), ciprofloxacin (trade name Cipro), and fluoxetine (trade name Prozac) under equivalent conditions. These compounds contain functional groups distinct from those of PFAS, including aromatic rings, amines, and carboxylic acids. As shown in Figures S11–S13, the SER spectra of these compounds exhibit distinct features that differ from those of the six targeted PFAS (Figure 2d), which consist of linear perfluoroalkyl chains, particularly in the CF_2_ and CF peak regions.^34–36^ These results suggest that SERS enables discrimination of PFAS from other structurally similar fluorinated species based on characteristic vibrational fingerprints. In addition, PFAS, with their linear perfluoroalkyl chains, exhibit strong hydrophobic and van der Waals interactions that promote adsorption onto the Au surface.^37^ In contrast, the more polar pharmaceutical molecules interact differently due to their aromatic rings, amines, and carboxylic groups, leading to distinct binding modes.^38^ These differences in surface affinity further contribute to the observed spectral distinctions and support the specificity of our SERS platform.

Quantitative analysis of the SER spectra was performed to see whether PFCAs with different chain lengths could be differentiated. Figure S14 shows how the Raman spectra positions associated change with respect to chain length, focusing on the alkyl chain C–C stretching vibrations (250 cm^–1^), the internal silicon standard (520 cm^–1^), and the C–F stretching bands (750 cm^–1^). The observed peak shifts exhibit a clear chain-length dependence with increases in the frequency gap (Δ*s*tandardized to Si) from C7 to C10. These spectral trends likely arise from enhanced vibrational coupling along the extended perfluoroalkyl backbone, as well as from increased van der Waals interactions between CF_2_ units for longer chain lengths. Furthermore, a similar trend is observed for the C–C symmetric stretching mode (1150 cm^–1^), highlighting the sensitivity of the SER spectra to small structural variations.^39^ These results confirm that SERS can resolve fine vibrational differences among structurally similar PFAS, enabling the discrimination of individual structural analogues based on their spectral fingerprints. Overall, these data suggest that the 6 PFAS can be quantified and differentiated using SERS at low concentrations.

To better understand the utility of SERS in detecting PFAS in real-world samples, we selected a commercial AFFF sample for quantification. Commonly used in firefighting, AFFFs are designed to rapidly spread across the surface of a flammable liquid, forming a thin aqueous film that separates the fuel from the oxygen in the air. To assess the PFAS content of the AFFF we designed a strategy to compare with the SERS analysis using ^19^F nuclear magnetic resonance (NMR) spectroscopy and liquid chromatography-tandem mass spectrometry (LC-MS/MS).

SERS was performed similarly to the measurements performed above, and the AFFF was drop-cast onto the concave cube substrate. Prominent features at 750 cm^–1^ (CF_2_ symmetric stretch) and 1375 cm^–1^ (CF stretching mode) were observed, which showed the possibility of containing perfluoro-carboxylic acid, such as PFOA and PFHpA; additional features near 1050 and 1150 cm^–1^ were assigned to the SO_3_ functional group stretching modes (Figure 3a). In comparison with the SERS of 10 ppm of PFOS (Figure 2), the functional group vibration peaks shown in PFOS are found in AFFF Raman spectra. Notably, these features are slightly blue-shifted in comparison, which we putatively ascribe to an additional additive to multi-PFAS, such as PFBuA and PFHxA in the AFFF complex mixture based on LC-MS/MS. As the carbon number of PFAS, the CF and CF_2_ peaks are slightly blue-shifted.^40^ These could modify the adsorption geometry of PFOS, altering its vibrational profile. Notably, there are also other features observed in the AFFF SER spectrum that are challenging to identify due to the proprietary nature of the mixture. Generally, AFFFs contain a mixture of water, hydrocarbon-based surfactants, and fluorochemical surfactants, all of which can have Raman-active vibrations.^7,24,41^ We note, however, that SERS clearly distinguishes the primary features of interest in the fluorinated PFAS backbone.

**Figure 3:**
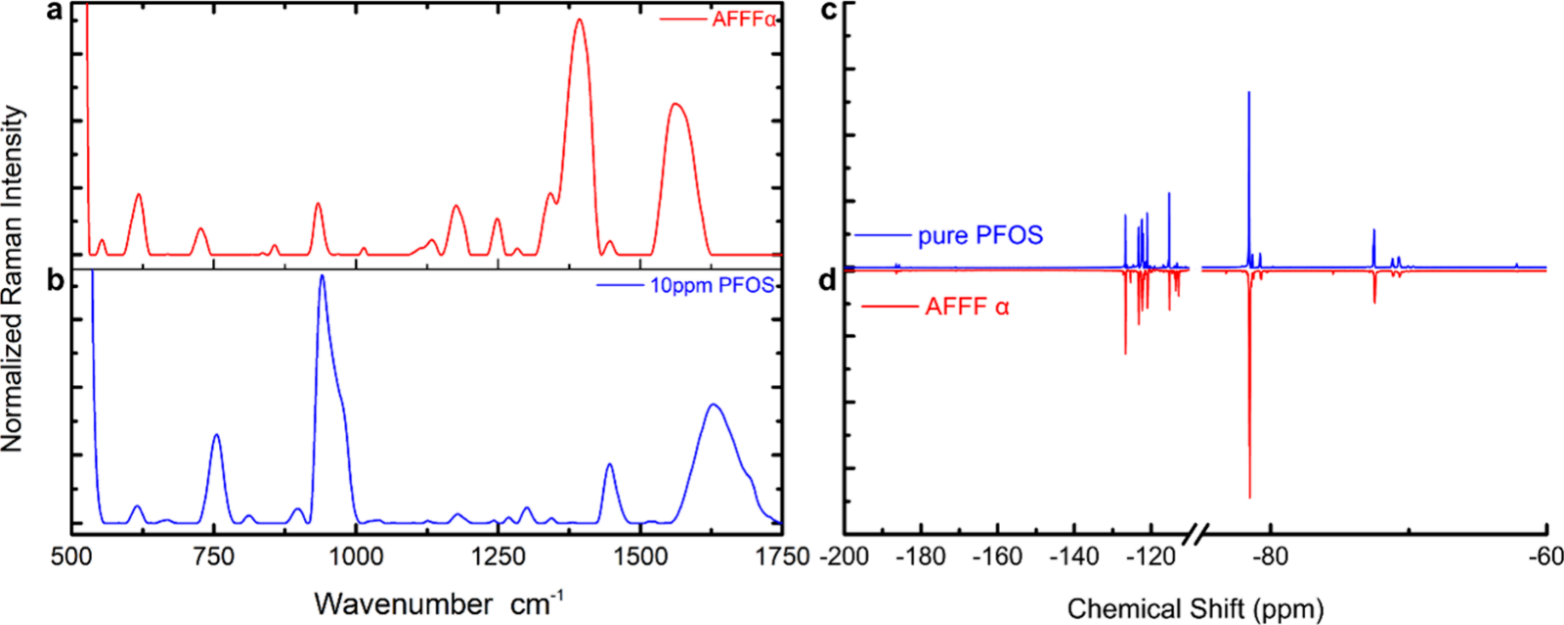
SERS and ^19^F NMR analyses of a commercial AFFF firefighting formulation. SER spectra of the AFFF (a) and 10 ppm PFOS (b). ^19^F NMR spectra of AFFF (c) and PFOS diluted in deuterated acetone (d).

AFFF samples and pure PFAS standards were dissolved in deuterated acetone in preparation for ^19^F NMR.^42,43^ NMR spectra were collected using a relaxation delay time of 4 s and 128 scans. Spectra of the six MassDEP-regulated PFAS were collected (Figure S2) and compared with the AFFF NMR spectrum (Figure 3c). After comparison with the pure standard compounds, ^19^F NMR suggests that PFOS is the major PFAS in this AFFF formulation (Figure 3d). Both NMR spectra of AFFF and PFOS show the terminal alkyl-CF_3_ fluorine nuclei at −82 ppm as well as the alkyl-CF_2_ fluorine nuclei that are adjacent to the sulfonic acid group near −115 ppm. The resonant peaks between −126 and −120 ppm are attributed to the remaining CF_2_ fluorines.^42–48^

To further validate the accuracy and feasibility of the application method, we prepared two additional AFFF foams: one PFAS-containing foam and the other one PFAS-free. The fluorine-free (PFAS-free) firefighting foam is primarily composed of hydrocarbon-based anionic and nonionic surfactants, biodegradable organic stabilizers, and solvents, without any added PFAS. These foams suppress fire by forming a stable aqueous blanket that cools the fuel and inhibits vapor release rather than by spreading a fluorinated film. Simultaneous measurements were taken using SERS and ^19^F NMR (Figure S6). NMR analysis verified the presence of PFAS species in the AFFF β sample. Meanwhile, the Raman spectra showed distinct characteristic peaks at around 750 and 1375 cm^–1^, further supporting the identification of PFAS in the sample. Both the ^19^F NMR and SER spectra of the AFFF β sample displayed key signatures indicative of the presence of both perfluoroalkyl carboxylic acids and perfluorosulfonic acids. Specifically, in the Raman spectra, strong peaks near 750 and 1375 cm^–1^ correspond to the symmetric CF_2_ and CF stretching vibrations characteristic of perfluoroalkyl chains, while additional peaks near 1050 and 1150 cm^–1^ are assigned to the SO_3_^–^ symmetric and asymmetric stretching modes, respectively, which are typical of sulfonic acid groups (Figure 3a). These vibrational features closely match those observed in the SERS spectra of the PFOS and PFOA standards (Figure 2). In addition, the ^19^F NMR spectra showed resonances at −82 ppm (CF_3_), −115 ppm (CF_2_ adjacent to sulfonic acid), and −126 to −120 ppm (remaining CF_2_ units), which collectively suggest the presence of PFOS-like and PFOA-like species. Taken together, these spectral features confirm that the AFFF formulation contains both perfluoroalkyl carboxylic acids and perfluorosulfonic acids. In contrast, the AFFF δ sample without PFAS did not exhibit these characteristic peaks in the Raman spectrum, and no PFAS was detected in the NMR analysis.

To independently quantify the PFAS content of the AFFF, LC-ESI-MS/MS was used (Table 1).^49,50^ The parameters of LC-ESI-MS/MS are detailed in Table S7 and the Methods. The MS data suggest that PFOS is the primary component of the 47 measured PFAS species in this commercial AFFF. When taken together, both LC-ESI-MS/MS and NMR have confirmed that PFOS is the primary component in AFFF. Moreover, they support the identification of PFAS by SERS in the complex AFFF matrix. Specifically, SERS can be used as a sufficient tool to confirm the presence of PFAS in a commercial product as well as to identify major PFAS species in solutions. A larger set of AFFF commercial mixtures is being used to validate the ability of SERS to confirm the presence of PFAS in such mixtures and to identify major PFAS species in them. Such a comparison is needed to validate the current observations and to generate more broadly applicable conclusions on the utility of SERS to screen for the presence of PFAS in commercial products.

**Table 1:** LC-ESI-MS/MS Results of PFAS Compounds in AFFF Sample Solution Mixture

PFAS (ng/mg)												
FOAM	PFBuA	PFHxA	PFHxS	PFHpA	PFOA	PFNA	PFOS	PFDA	GenX	PFUdA	Me-FOSAA	Et-FOSAA
AFFF	238	430		93.0	396		24,200		36.2			
limit of detection	0.7	0.2	0.2	0.2	0.2	0.1	0.7	0.5	3.0	0.5	5.0	4.0

## Conclusion

We have shown that PFAS can be identified in complex commercial AFFF mixtures using rapid SERS-based detection. Concave cubic nanoparticle SERS substrates excited at 785 nm were found to detect the 6 PFAS regulated by MassDEP at parts per million levels, while identifying PFOS in AFFF complex mixtures. Spontaneous bulk Raman and DFT-calculated spectra specifically identified CF_2_ symmetric stretches and CF stretching modes as the key vibrations for PFAS identification. ^19^F NMR and LC-MS/MS were separately used to analyze and confirm the presence of PFOS in the unknown AFFF matrix. These results highlight how SERS provides a means for rapid nondestructive detection of PFAS in complex mixtures, potentially providing a means for rapid screening of complex mixtures for fluorinated compounds. This capability has important implications for the analysis of hazardous waste, water runoff, and other PFAS-containing media. Future efforts should focus on improving the durability of SERS substrates to reduce interference from complex foam matrices and environmental samples while also lowering detection limits to enhance sensitivity. As part of our ongoing research, we are actively designing experiments to include a broader group of PFAS analogues with different head groups and ether linkages to evaluate the platform’s resolving power in more complex mixtures. This future work will be critical for validating the method’s capability to distinguish and quantify all six PFAS regulated by MassDEP and their structurally related analogues in real-world matrices. Advancing SERS capabilities to reliably identify PFAS in complex mixtures is essential for their broader application in real-world environmental monitoring. Additionally, a more comprehensive analytical approach is needed to clarify the diverse additives in AFFF formulations, and expanding the range of PFAS compounds investigated will be critical for addressing the complexity of environmental contamination and ensuring effective detection strategies.

## Materials and Methods

### Chemicals and Reagents

Pentadecafluorooctanoic acid (PFOA > 98.0%), heptadecafluorononanoic acid (PFNA > 95.0%), nonadecafluorodecanoic acid (PFDA > 98.0%), tridecafluoroheptanioc acid (PFHpA > 98.0%) from TCI, tridecafluorohexane-1-sulfonic acid potassium salt (PFHxS ≥ 98%), heptadecafluorooctanesulfonic acid potassium salt (PFOS-K ≥ 98%), and cetyltrimethylammonium chloride solution (CTAC, 25 wt% in H_2_O, Lot No. STBK3589) were purchased from Sigma-Aldrich. Hydrogen tetrachloroaurate trihydrate (HAuCl_4_·3H_2_O, ACS, 99.99% (metal basis), Au 49.0% min), Silver nitrate (AgNO_3_, Premion 99.9995% (metal basis)), Sodium borohydride (NaBH_4_, 97 + % −10 + 40 Mesh granules) from Alfa Aesar, Hydrochloric acid (HCl, A142-212, Technical grade) from Fisher Chemical, and l-ascorbic acid (ACS reagent, ≥99 + %), were purchased from Thermofisher Scientific. Deuterated Acetone (Acetone-d6, for NMR, 99+ atom% D) from Acros Organics. All solutions were prepared with deionized water (18.2 MΩ resistivity, Milli-Q). A commercially available AFFF formulation (FC-201F, light water 1% concentrate, 3M) was acquired directly from a container at a fire training facility.

### Concave Cubic Gold Nanoparticle Synthesis

Synthesis of Au seeds: 0.250 mL of 10 mM HAuCl_4_ and 0.02 mL of 1 M HCl were added sequentially to 10.0 mL of 100 mM CTAC with vigorous stirring, resulting in a light-yellow solution. Next, 0.60 mL of freshly prepared 10 mM NaBH_4_ was rapidly injected into the vigorously stirring solution, which led to the formation of an orange-brown seed solution. The seed solution was allowed to stir for an additional minute to ensure uniform dispersion of the reducing agent. The solution was allowed to age at room temperature for 2 h.

Next, 0.20 mL of 1 M HCl, 0.50 mL of 10 mM HAuCl_4,_ and 100 μL of 10 mM AgNO_3_ were added sequentially into 10.0 mL of 100 mM CTAC with gentle swirling. Then 0.10 mL of 100 mM ascorbic acid was introduced, causing a noticeable color change from light yellow to clear. 0.10 mL of an aliquot of seeds, which had been diluted 1000× in 100 mM CTAC, was then added to initiate the growth of particles.

### Density Function Theory Calculation

Raman spectrum calculations in this work were performed using the GaussView computational software. Geometry optimization, frequency, and Raman polarization were calculated by setting up the method of ground-state density function theory (DFT) calculation with the B3LYP model. The valence orbital energy and binding vibrational calculations used 6–31G­(d,p) as a “double-ζ” approach.

### NMR Spectroscopy

All NMR samples were directly prepared in NMR tubes. All NMR experiments were recorded on a Joel EZC 400 spectrometer with a variable temperature unit. The data were processed with Delta 6.0 software. Standard ^19^F NMR spectra were recorded at 400 MHz.

### Fourier Transform Infrared Spectrometer

Fourier transform infrared spectroscopy (FTIR) data were recorded from a Jasco ATR FT/IR-6600, with a DLaTGS detector, scanning wavenumber range from 400 to 4000 cm^–1^, a scanning speed of 2 mm/s, a resolution set to 4 cm^–1^, and an accumulation time of 20 s.

### SERS Scan of PFAS

SERS data were collected from a Bruker Senterra Dispersive Raman microscope, Serial number R200.0238, Detector model DU420A-OE-152, at a laser 785 nm. To avoid damage due to laser-induced heating, all measurements were performed at a low laser power of 50 mW. The integration time was set to 10 s, with an averaging of 4 scans to improve the signal-to-noise ratio. Concave cube Au nanoparticles were synthesized by following the method above, transferred to a 2 mL centrifuge tube, and concentrated to 50 μL. The concentrated nanoparticle solution was drop-cast onto a silicon wafer and dried at room temperature. SERS PFAS samples were prepared by diluting 6-MA-regulated PFAS stock solutions to different low concentrations down to 0.1 ppm. A silicon wafer with a concave cube Au nanoparticle was placed on a glass slide, a drop-cast-diluted PFAS solution was placed on the silicon wafer surface, and dried at room temperature.

The frequency gap (Δ) was calculated from Raman spectra as the difference between the C–C alkyl chain stretching, located near 250 cm^–1^, and the C–C symmetric stretching modes, located near 1150 cm^–1^, referenced to an internal silicon standard, located at 520 cm^–1^, and normalized to the characteristic C–F stretching vibration, located near 750 cm^–1^.

### Scanning Electron Microscopy

SEM images were taken on a JSM-7401F field-emission scanning electron microscope at 10 kV electron voltage with 10 μA emission current under 40k magnification. A TEM image was taken on a Philips CM-12 at a high-tension voltage of 120 kV under 45k magnification.

### Liquid Chromatography–Negative Electrospray Ionization-tandem-mass Spectrometry

PFAS species were quantified by liquid chromatography–negative electrospray ionization-tandem mass spectrometry (LC-ESI-MS/MS) in an Applied Biosystems API4000 triple quadrupole mass spectrometer (Sciex, MA) using the isotope dilution method. The experiments were performed in negative ionization mode. The source conditions (collision gas flow, curtain gas flow, ion source gas flow, and ion spray voltage) were optimized for maximum sensitivity (Table S7). Chromatographic separation was accomplished on a Shimadzu LC20 series stack using a Luna Omega PS C18, 3 μm, 100 Å, 100 × 4.6 mm (Phenomenex). Mobile phases were 10 mM ammonium acetate in D.I. water (A) and 10 mM ammonium acetate in methanol (B). The chromatographic gradient was 10% B in the first minute, to 65% B at 2 min, to 99% B at 15 min, holding 99% B to 20 min, followed by 5 min postcolumn equilibration. The sample injection volume was 10 μL. Background PFAS contamination was eliminated using an online delay column (Ascentis Express 160 Å FAS Delay, 2.7 μm, 50 × 4.6 mm). A diverter valve (VICI, Valco Instrument Co. Inc.) was used to divert the first 3 min and the last 8 min of the chromatographic run to waste. The scheduled MRM mode (Table 1) was used for data acquisition of the target set of PFASs. Twenty % of the samples were analyzed as true blind duplicates.

## Supplementary Material



## Notes

Supporting Information contains additional Raman spectra, analysis, control experiments, and DFT geometries (PDF)
