# Supplementary material for: Surface-Enhanced Raman Spectroscopy Detection of Per- and Polyfluoroalkyl Substances in Aqueous Film-Forming Foams
Source: Environ Sci Technol. 2025 Dec 21;60(1):1153–60. doi: 10.1021/acs.est.5c10183 (PMC12810368; doi:10.1021/acs.est.5c10183)
Supplement: Supplementary file 1 [file es5c10183_si_001.pdf]

Supporting Information for:

Surface-Enhanced Raman Spectroscopy Detection of Per- and Poly-Fluoroalkyl Substances in Aqueous Film Forming Foams

Chuntao Wang,[1] Kushal Biswas,[2] Sangmin Jeong,[1] Anila Bello,[3] Dhimiter Bello,[2] Michael B. Ross[1]*

[1] Department of Chemistry, University of Massachusetts Lowell, Lowell, MA, USA 01854

[2] Department of Biomedical and Nutritional Science, University of Massachusetts Lowell, Lowell, MA, USA 01854

[3]Department of Public Health, University of Massachusetts Lowell, Lowell, MA, USA 01854

*Email: Michael_ross@uml.edu

Table of Contents

S2: FTIR and NMR spectra of PFAS

S3 FTIR spectra of AFFF; SERS control spectra and analysis

S4 Concentration dependent SER spectra of PFOS

S5 SER and ^{19}F NMR spectra of AFFFs; Water control spectra

S6 SER spectra at 532 nm; SERS PFOA calibration curve

S7 SERS PFOS calibration curve; Spectra of atorvastatin; Spectra of fluoxetine

S8 Spectra of ciprofloxacin; PFCA chain length analysis by SERS

S9-S20 DFT geometries and vibrational tables

S21 LC-ESI-MS/MS analysis of AFFF

S22 Total fluorine analysis of AFFF

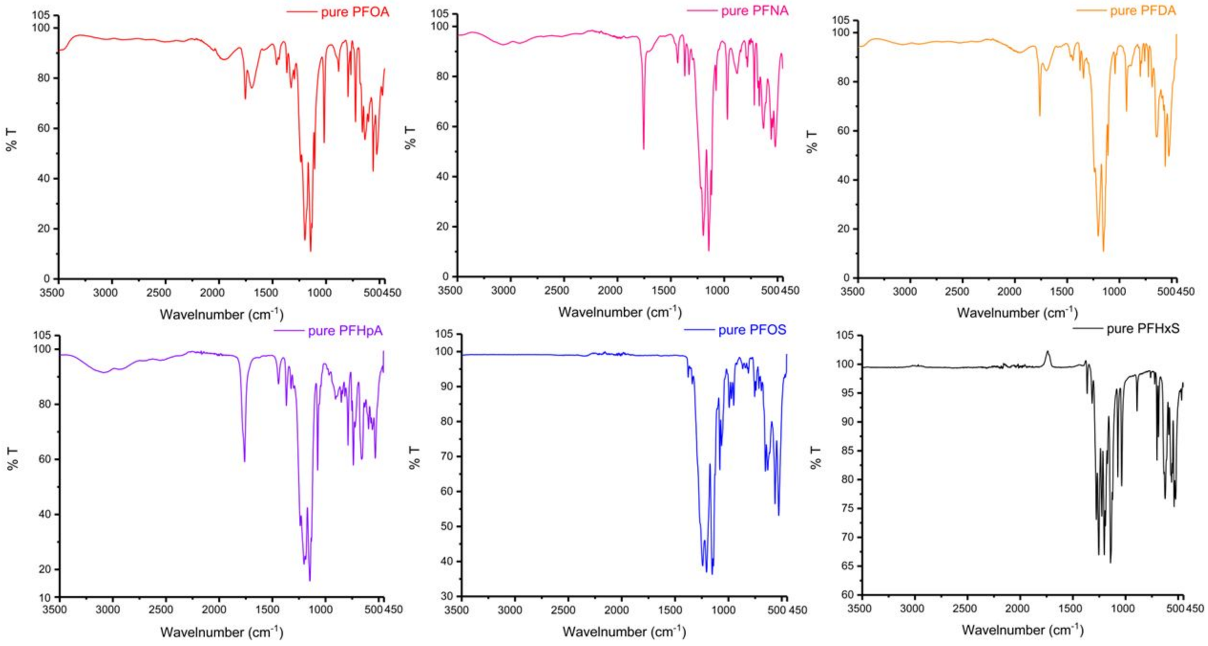

Figure S1. FTIR spectra of 6 Massachusetts drinking water regulated PFAS: PFOA (red), PFNA (pink), PFDA (orange), PFHpA (violet), PFOS (blue), PFHxS (Black)

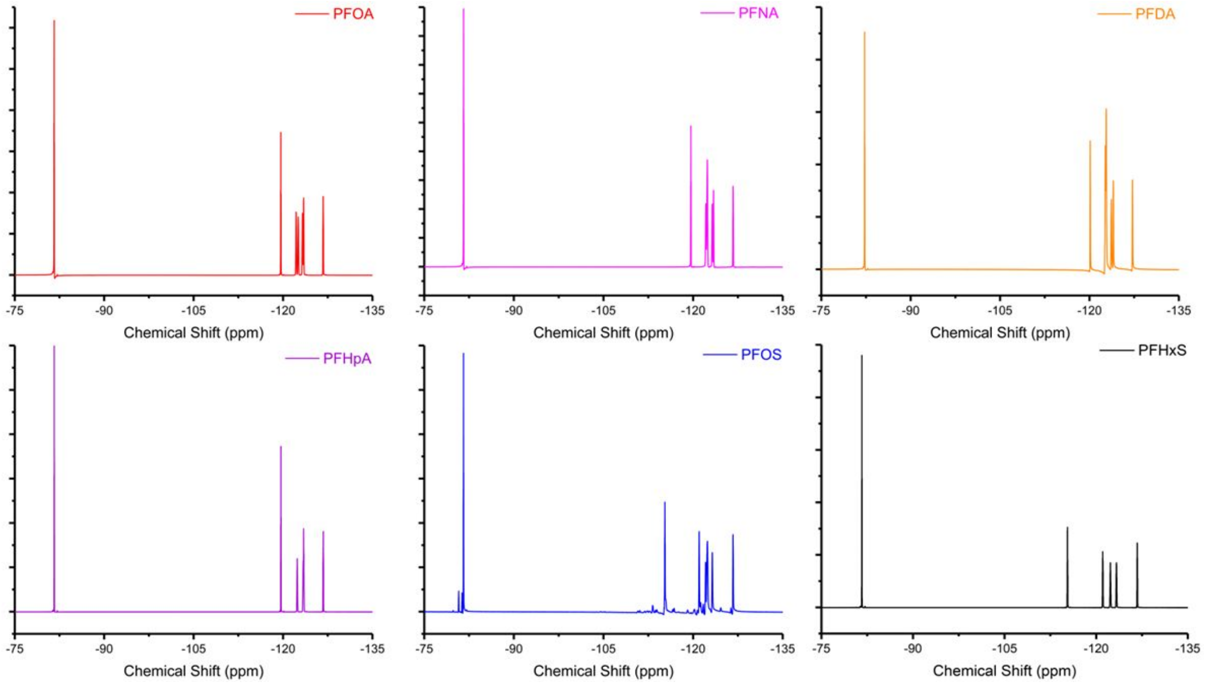

Figure S2. NMR spectra of 6 Massachusetts drinking water regulated PFAS: PFOA (red), PFNA (pink), PFDA (orange), PFHpA (violet), PFOS (blue), PFHxS (Black)

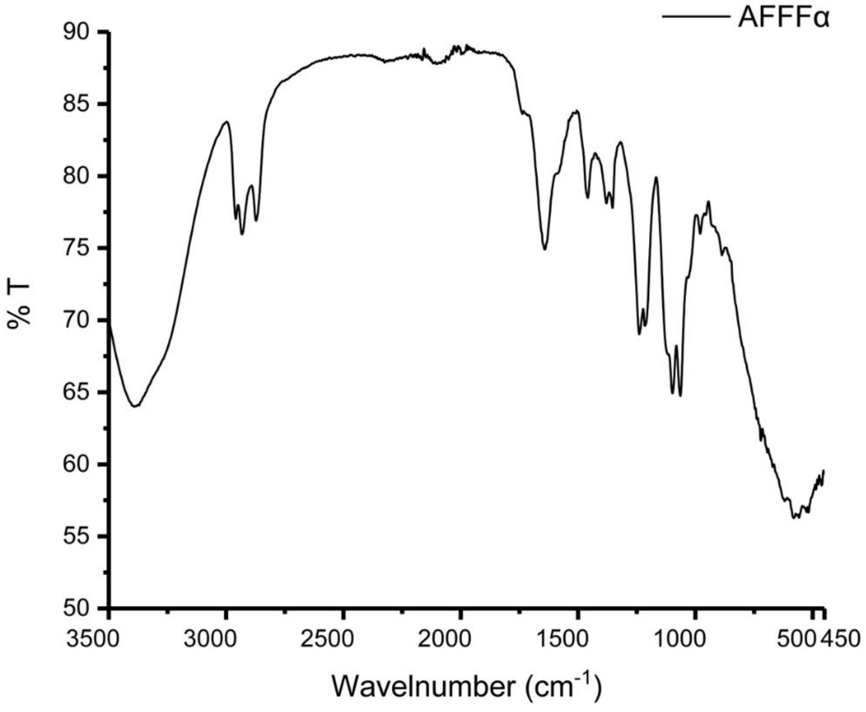

Figure S3. FTIR spectrum of Aqueous Film Forming Foam (AFFFα) sample

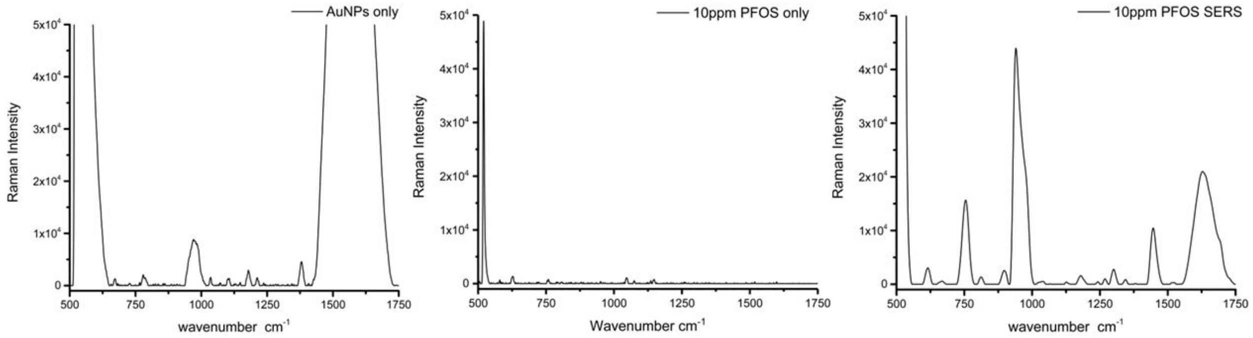

Figure S4. SERS spectra of AuNPs only, 10 ppm PFOS without AuNPs, and 10 ppm PFOS on Si wafer with AuNPs

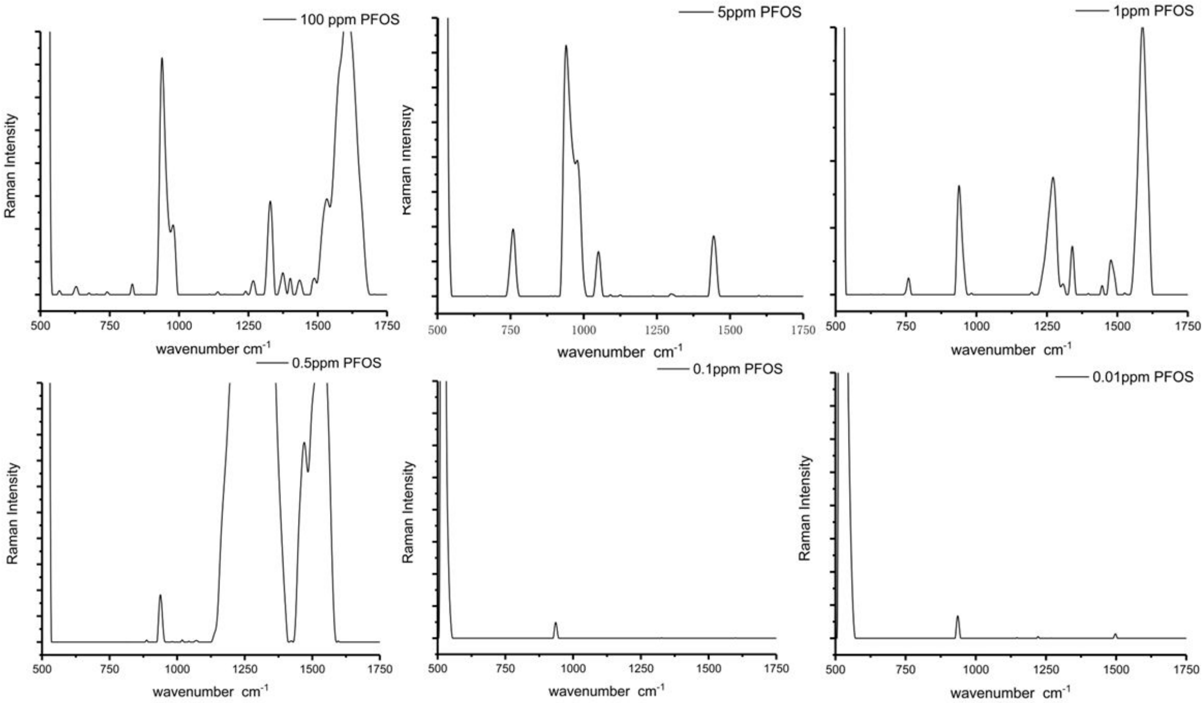

Figure S5. SERS spectra of PFOS solutions at different concentrations 0.01ppm, 0.1ppm, 0.5ppm, 1ppm, 5ppm, 100ppm.

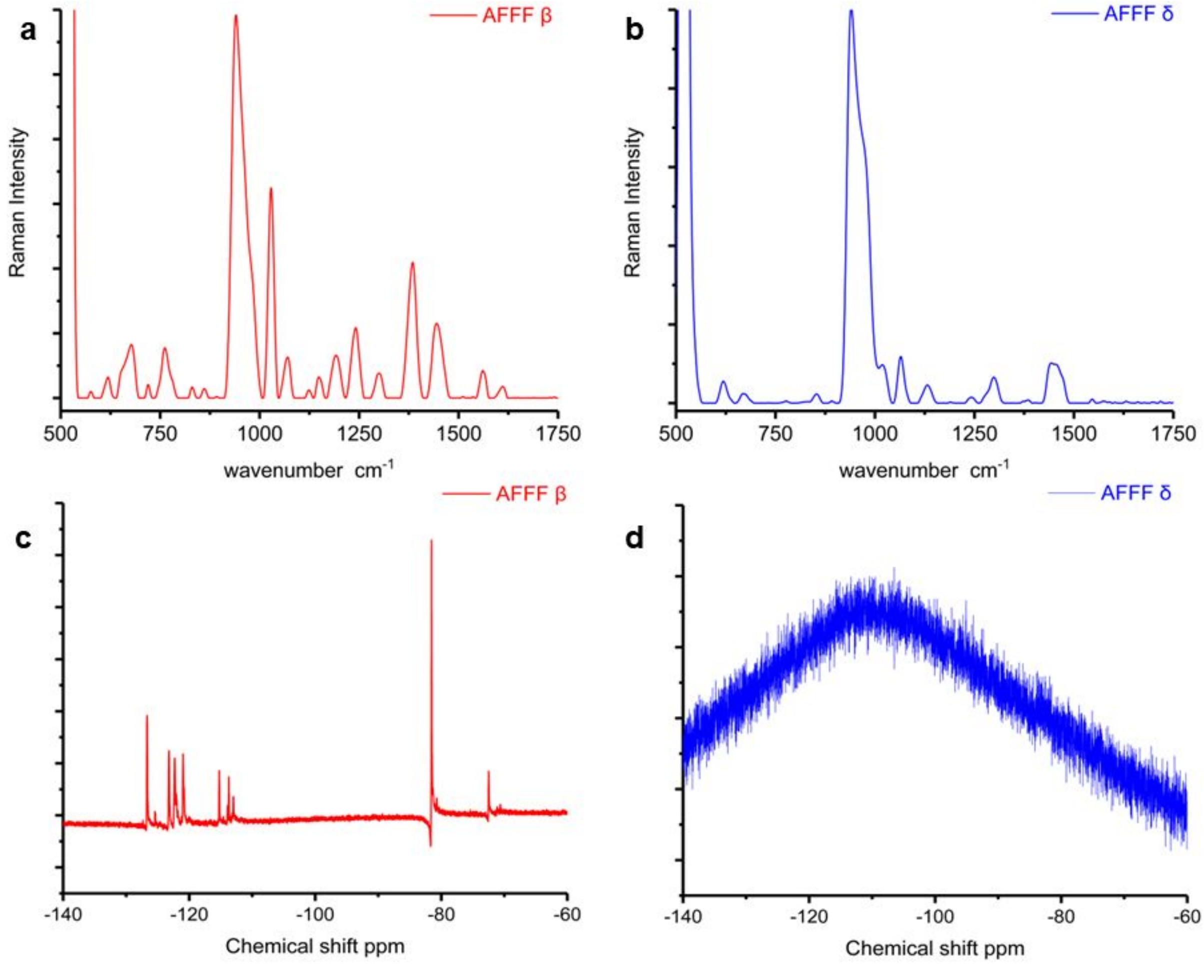

Figure S6. Graph a & b are SERS spectra of AFFF β and AFFF δ. Graph c & d are ^{19}F NMR spectra of AFFF β and AFFF δ.

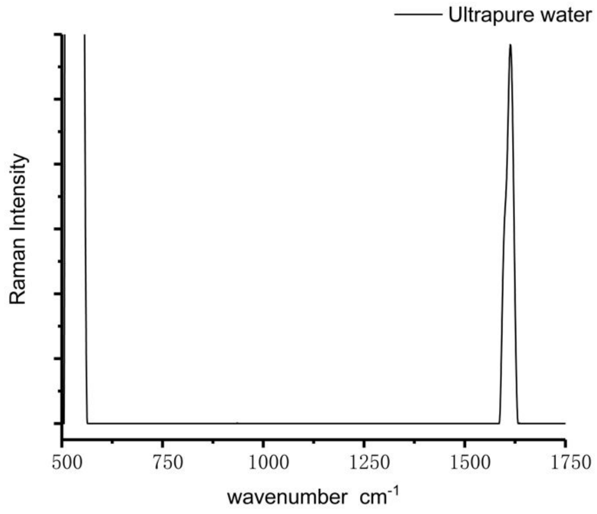

Figure S7. SERS spectrum of ultrapure water only dropcast on AuNPs decorated Si wafer.

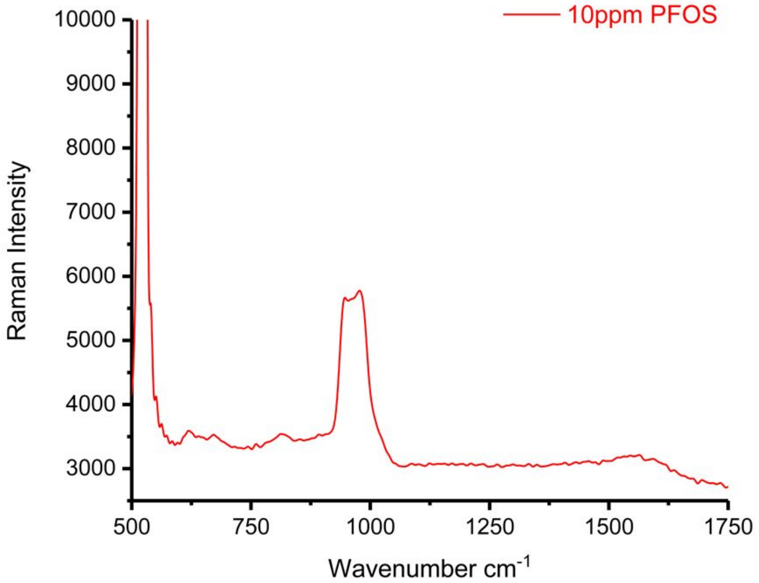

Figure S8. SERS spectrum of 10ppm PFOS on AuNPs decorated Si-wafer under laser wavelength 532 nm

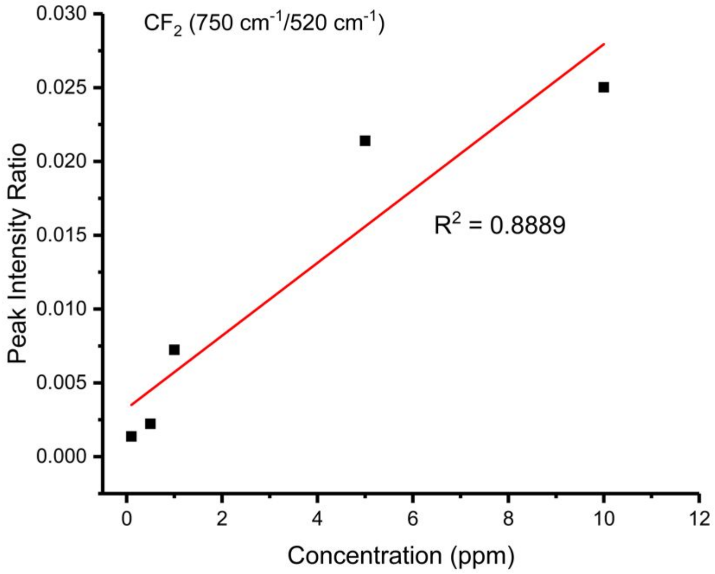

Figure S9. Standard Calibration Curve of PFOA range from 0.1ppm to 10ppm

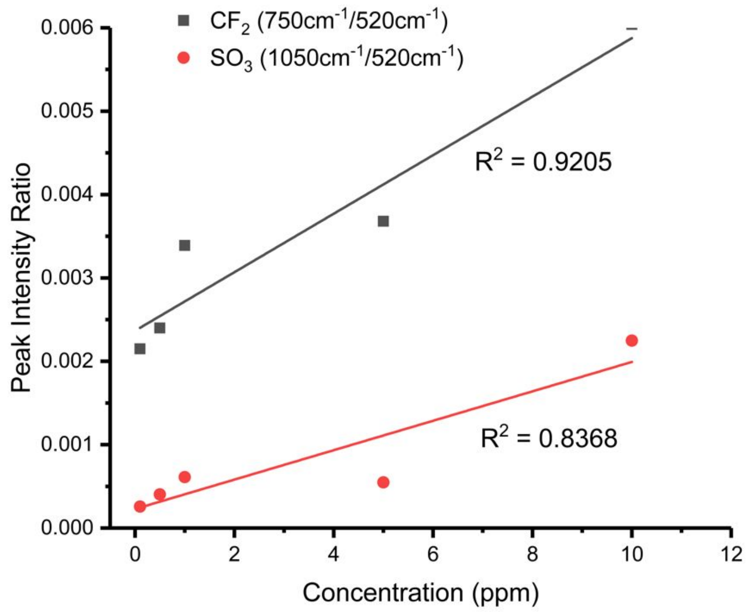

Figure S10. Standard Calibration Curve of PFOS range from 0.1ppm to 10ppm

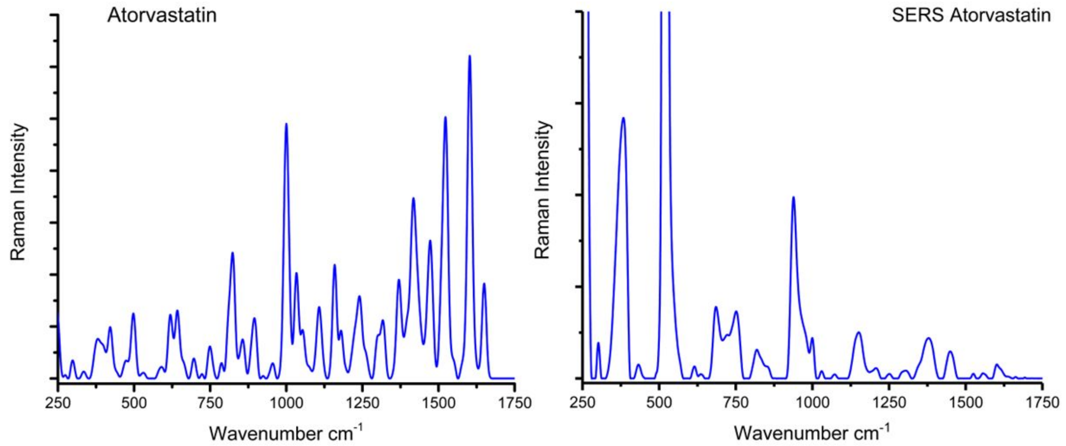

Figure S11. Raman spectrum of Atorvastatin and SER spectrum of Atorvastatin

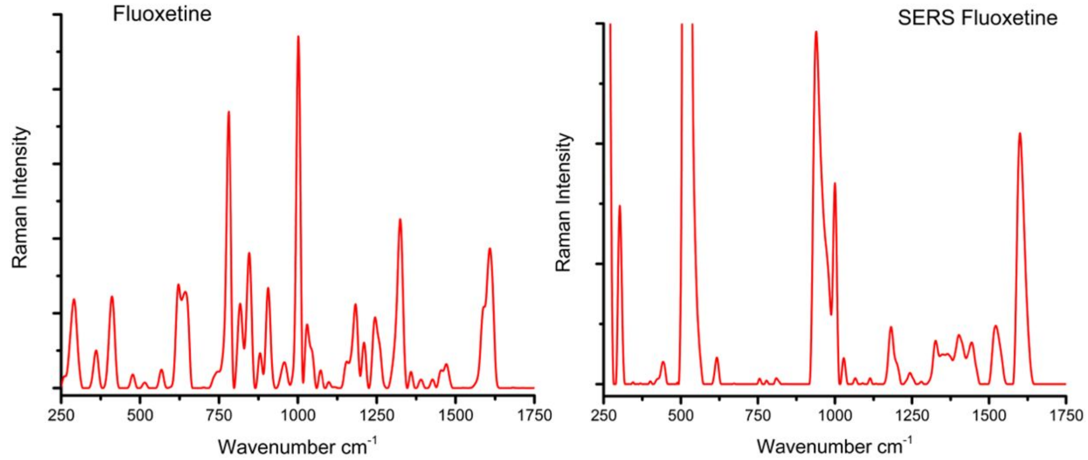

Figure S12. Raman spectrum of Fluoxetine and SER spectrum of Fluoxetine

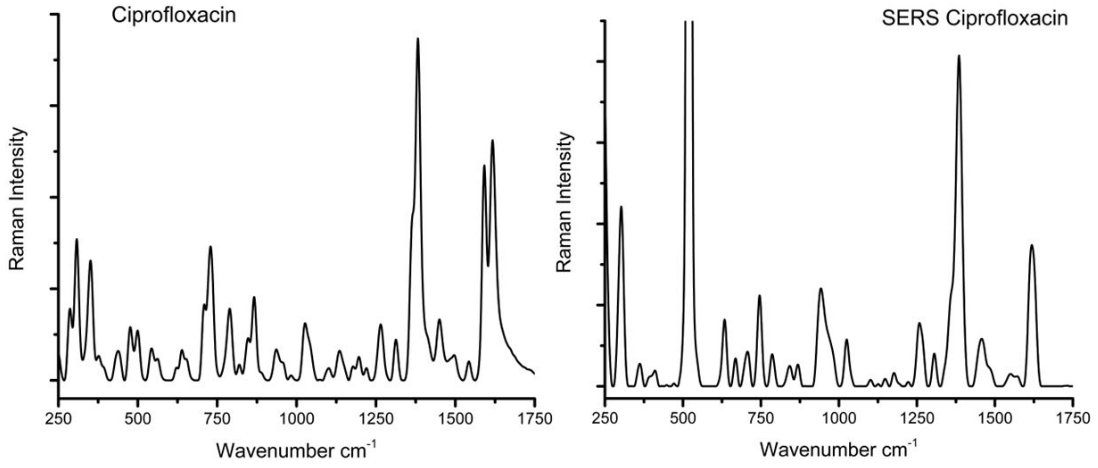

Figure S13. Raman spectrum of Ciprofloxacin and SER spectrum of Ciprofloxacin

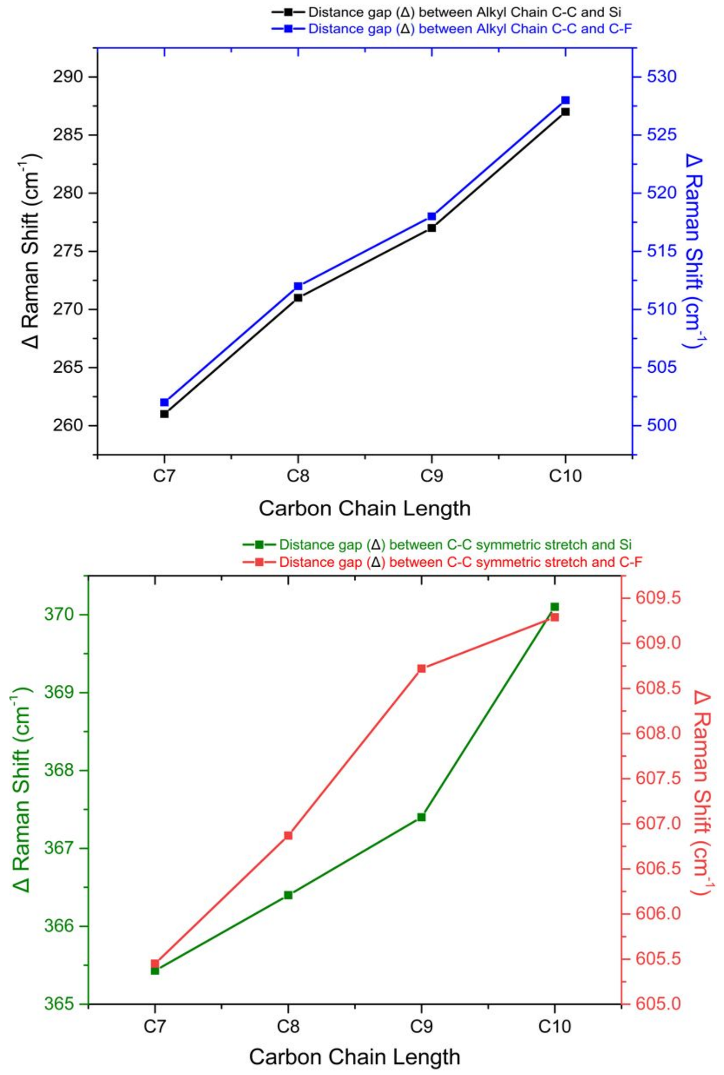

Figure S14. Raman peak position distance gap (Δ) between the C–C stretching mode and the Si reference peak, as well as between the C–C and C–F vibrational modes.

Table S1 DFT calculation data of vibration mode of PFDA

Mode #	Frequency cm^{-1}	Infrared Intensity	Raman Activity	Depolar-P	Depolar-U
1	21.29	0.0961	0.0461	0.736	0.8479
2	22.45	0.4723	0.2165	0.7432	0.8527
3	27.72	0.142	0.0212	0.749	0.8565
4	32.71	1.46	0.8113	0.7487	0.8563
5	41.32	0.4822	0.1009	0.7471	0.8552
6	47.85	0.1759	0.0027	0.7421	0.8519
7	53.95	0.0757	0.0558	0.7412	0.8514
8	58.59	0.0561	0.0255	0.7486	0.8562
9	64.23	0.046	0.0009	0.7358	0.8478
10	68.02	0.0316	0.0111	0.7392	0.8501
11	95.21	0.1618	0.2842	0.5547	0.7136
12	111.96	0.0034	1.1655	0.2073	0.3434
13	133.85	0.2792	0.1525	0.7485	0.8561
14	170.39	0.4864	0.0814	0.6237	0.7682
15	181.72	0.4742	0.0192	0.703	0.8256
16	195.73	6.2954	0.1401	0.7498	0.857
17	204.12	1.5087	0.0096	0.5919	0.7437
18	208.04	0.3365	0.0476	0.6895	0.8162
19	214.83	0.0723	0.0357	0.42	0.5916
20	219.79	0.348	0.0082	0.4868	0.6548
21	233.27	0.0588	0.0322	0.7224	0.8388
22	244.15	0.0584	0.0516	0.75	0.8571
23	244.42	0.0304	0.0093	0.5158	0.6806
24	254.49	0.9878	0.0905	0.655	0.7915
25	259.18	0.1119	0.0332	0.2673	0.4218
26	261.28	0.1963	0.0273	0.7156	0.8342
27	288.76	5.6411	0.9502	0.35	0.5186
28	299.66	0.1274	2.3468	0.7465	0.8549
29	304.03	0.309	3.3006	0.7478	0.8557
30	323.43	0.1094	0.0597	0.7049	0.8269
31	331.43	0.7037	0.3488	0.6135	0.7605
32	347.5	0.1296	0.5442	0.7078	0.8289
33	356.79	0.7493	0.2081	0.7397	0.8504
34	365.27	0.1155	0.073	0.701	0.8242
35	367.76	0.0806	0.4215	0.3603	0.5297
36	369.71	2.6662	1.8458	0.3246	0.4902
37	372.05	1.4051	1.0598	0.75	0.8571
38	377.19	0.3889	1.852	0.2181	0.3581
39	380.86	0.4468	0.4545	0.5972	0.7478
40	421.64	0.3876	0.3089	0.6776	0.8078

41	464.81	1.1575	0.0597	0.4416	0.6126
42	505.15	11.7394	0.3883	0.614	0.7609
43	510.04	88.9677	0.6936	0.5507	0.7102
44	523.84	6.3378	0.9998	0.4143	0.5858
45	545.23	15.2809	0.1711	0.575	0.7302
46	545.84	28.941	0.0692	0.75	0.8571
47	559.85	84.2996	0.6525	0.749	0.8565
48	563.17	14.8933	0.8465	0.6886	0.8156
49	579.43	14.7824	0.639	0.7093	0.8299
50	597.05	3.3394	2.3804	0.7274	0.8422
51	601.5	22.8717	1.4277	0.6831	0.8117
52	617.21	22.689	0.9346	0.6585	0.7941
53	637.79	185.2832	1.4286	0.6828	0.8115
54	655.36	154.5424	2.7508	0.6691	0.8017
55	670	14.1829	0.6797	0.5007	0.6673
56	688.72	13.8766	3.1587	0.1355	0.2387
57	707.38	6.2902	0.1827	0.5892	0.7415
58	725.82	1.7991	11.6386	0.0074	0.0147
59	743.6	5.7224	1.7186	0.112	0.2014
60	768.71	23.907	9.3415	0.0554	0.105
61	815.17	4.208	1.4563	0.0946	0.1728
62	935.56	51.0312	0.435	0.6322	0.7747
63	1047.03	8.0482	0.8651	0.1576	0.2723
64	1119.84	32.9388	0.1467	0.7491	0.8565
65	1142.6	10.1918	0.1077	0.0908	0.1665
66	1153.34	23.5902	0.2854	0.3947	0.566
67	1163.74	186.2172	0.5682	0.3151	0.4792
68	1177.45	174.8242	0.3319	0.6277	0.7713
69	1185.41	97.3574	1.4786	0.6503	0.7881
70	1193.63	6.7916	0.0915	0.5401	0.7014
71	1197.81	68.2509	0.2569	0.7488	0.8563
72	1201.62	7.3244	0.5561	0.7458	0.8544
73	1206.13	96.7782	2.4994	0.6547	0.7913
74	1219.11	106.8484	0.5399	0.5554	0.7142
75	1221.84	11.512	1.2593	0.7371	0.8486
76	1223.43	134.3391	1.2201	0.6808	0.8101
77	1227.66	3.263	1.0304	0.7357	0.8477
78	1238.08	162.0905	0.3971	0.466	0.6357
79	1247.08	553.0565	0.5872	0.6687	0.8015
80	1260.31	159.9628	1.0444	0.6278	0.7713
81	1269.9	339.1458	0.515	0.6061	0.7547
82	1272.94	182.2409	1.5437	0.7404	0.8509
83	1297.91	54.3091	0.6399	0.6757	0.8065

84	1313.8	29.0124	3.5609	0.7279	0.8426
85	1319.33	0.6697	6.247	0.7212	0.838
86	1346.38	41.4769	9.0029	0.6555	0.7919
87	1384.63	61.7446	17.3304	0.7475	0.8555
88	1415.23	52.2268	6.2264	0.7486	0.8562
89	1874.63	251.9135	6.7734	0.3442	0.5121
90	3745.63	83.8013	138.7855	0.3021	0.464

Table S2, DFT calculation data of vibration mode of PFNA

Mode #	Frequency cm^{-1}	Infrared Intensity	Raman Activity	Depolar-P	Depolar-U
1	23.29	0.526	0.2488	0.7404	0.8508
2	25.04	0.2177	0.0463	0.7489	0.8564
3	32.22	1.1356	0.6399	0.7477	0.8557
4	34.98	0.4108	0.2178	0.7494	0.8567
5	42.8	0.3812	0.0946	0.7467	0.855
6	53.77	0.2499	0.0081	0.7487	0.8563
7	61.18	0.0394	0.0663	0.7291	0.8434
8	62.08	0.0068	0.0055	0.7448	0.8538
9	68.1	0.0595	0.0295	0.7264	0.8415
10	105.14	0.2602	0.1869	0.7232	0.8394
11	119.58	0.0287	1.3836	0.2533	0.4042
12	149.24	0.2398	0.1173	0.2017	0.3357
13	177.33	0.6111	0.1286	0.7344	0.8468
14	196.24	5.9795	0.1272	0.7494	0.8568
15	203.71	0.2512	0.0095	0.587	0.7398
16	206.97	1.0512	0.0015	0.5757	0.7307
17	211.56	0.3209	0.024	0.7288	0.8431
18	224	0.3774	0.006	0.7435	0.8529
19	238.26	0.0167	0.0179	0.7492	0.8566
20	242.3	0.0476	0.0552	0.7422	0.852
21	252.76	0.973	0.0641	0.4007	0.5722
22	257.77	0.0044	0.0026	0.5204	0.6845
23	259.73	0.2614	0.0894	0.7388	0.8498
24	288.75	5.6476	0.9853	0.3458	0.5139
25	301.67	0.1039	2.2368	0.7495	0.8568
26	306.16	0.061	3.1766	0.7425	0.8523
27	328.38	0.1515	0.0833	0.3613	0.5308
28	338.61	1.0741	0.1873	0.7498	0.857
29	353.29	0.1521	0.4772	0.7488	0.8564
30	361.49	0.4889	0.2025	0.4086	0.5802
31	366.75	0.0163	0.1875	0.2688	0.4237

32	370.36	0.4761	2.4868	0.4519	0.6225
33	371.36	3.6953	0.8798	0.1991	0.332
34	377.21	0.1136	1.766	0.3899	0.5611
35	392.4	0.5028	0.2114	0.3935	0.5648
36	439.57	0.5529	0.2789	0.6193	0.7649
37	486.72	1.2115	0.0931	0.7357	0.8478
38	508.83	70.2673	0.9871	0.5379	0.6996
39	524.07	9.5116	0.9792	0.4006	0.5721
40	532.77	8.618	0.1844	0.3968	0.5681
41	549.34	14.825	0.1596	0.4236	0.5951
42	557.42	80.5445	0.6191	0.7431	0.8526
43	567.81	4.8641	0.6366	0.746	0.8546
44	588	13.8795	1.2189	0.7343	0.8468
45	597.28	29.9442	1.5174	0.679	0.8088
46	605.57	16.461	1.5952	0.662	0.7966
47	628.21	71.1369	1.2257	0.6862	0.8139
48	651.29	246.6983	4.0937	0.6644	0.7984
49	662.15	24.8805	0.0604	0.7249	0.8405
50	690.43	14.3177	3.1229	0.1727	0.2946
51	704.46	21.3331	0.6789	0.2988	0.4601
52	724.34	2.4505	11.4506	0.0075	0.0149
53	749.54	7.2168	1.4074	0.203	0.3375
54	771.3	21.5503	8.2	0.0552	0.1047
55	846.02	3.7212	1.4892	0.0573	0.1085
56	978.92	57.2203	0.6387	0.6894	0.8161
57	1088.12	6.3584	0.7718	0.1487	0.2589
58	1137.5	28.3809	0.0562	0.5603	0.7182
59	1150.54	1.604	0.3198	0.2231	0.3648
60	1162.06	188.631	0.5818	0.4186	0.5901
61	1176.1	182.4221	0.3602	0.3719	0.5421
62	1185.77	117.9594	1.6907	0.6564	0.7926
63	1195.46	2.3333	0.2811	0.6041	0.7532
64	1197.81	17.6224	0.6204	0.7493	0.8567
65	1205.86	28.669	0.8137	0.7466	0.8549
66	1209.41	20.3904	1.5316	0.706	0.8277
67	1219.91	215.8422	0.7458	0.7002	0.8237
68	1226.7	104.3268	1.8007	0.7156	0.8343
69	1231.57	111.7094	1.1483	0.7058	0.8275
70	1243.47	324.1292	0.2819	0.7178	0.8357
71	1245.2	92.9735	1.1859	0.4255	0.597
72	1255.91	160.2868	0.9413	0.7319	0.8452
73	1271.27	537.9308	1.0576	0.7073	0.8285
74	1289.92	50.1334	0.4763	0.6304	0.7733

75	1306.03	28.5195	1.8944	0.6429	0.7827
76	1317.19	12.5114	8.2228	0.7253	0.8408
77	1341.13	39.4187	7.7282	0.663	0.7973
78	1382.55	72.574	15.9835	0.7484	0.8561
79	1415.27	46.1462	6.144	0.7497	0.857
80	1874.63	252.0491	7.5666	0.3414	0.509
81	3745.63	83.5153	146.2029	0.3029	0.4649

Table S3, DFT calculation data of vibration mode of PFOA

Mode #	Frequency cm^{-1}	Infrared Intensity	Raman Activity	Depolar-P	Depolar-U
1	25.14	0.5225	0.3001	0.7412	0.8514
2	29.26	0.821	0.2758	0.7491	0.8565
3	35.34	0.9525	0.6061	0.7484	0.8561
4	37.41	0.2046	0.0324	0.7461	0.8546
5	48.57	0.1998	0.0385	0.7491	0.8565
6	58.6	0.1157	0.0031	0.7102	0.8306
7	63.98	0.0569	0.0198	0.7385	0.8496
8	70.98	0.1328	0.0848	0.7438	0.8531
9	113.66	0.3109	0.4398	0.5283	0.6914
10	135.9	0.0144	1.0978	0.1977	0.3301
11	159.07	0.3288	0.1679	0.75	0.8571
12	196.3	5.149	0.0857	0.7071	0.8284
13	201.29	1.1195	0.0603	0.5581	0.7164
14	206.04	0.264	0.0054	0.6628	0.7972
15	210.84	0.5845	0.006	0.6164	0.7627
16	229.4	0.3572	0.0046	0.6305	0.7734
17	238.57	0.0422	0.0468	0.7196	0.8369
18	250.44	0.689	0.0437	0.5391	0.7005
19	254.79	0.0638	0.0422	0.5847	0.7379
20	258.92	0.5374	0.0788	0.7144	0.8334
21	288.73	5.3428	0.9625	0.3505	0.5191
22	304.31	0.0771	2.0838	0.7394	0.8502
23	308.91	0.2463	2.8745	0.7471	0.8552
24	334.27	0.2337	0.0446	0.6935	0.819
25	347.51	1.236	0.1763	0.2584	0.4106
26	359.57	0.2203	0.4185	0.7495	0.8568
27	364.76	0.1622	0.0493	0.4056	0.5771
28	369.12	0.1424	1.4895	0.5331	0.6954
29	372.29	3.6165	1.85	0.2248	0.3671
30	376.73	0.1681	1.6241	0.4639	0.6338

31	408.44	0.5502	0.2257	0.4733	0.6425
32	461.55	0.9474	0.2346	0.7042	0.8264
33	506.76	32.7376	0.7487	0.6202	0.7656
34	517.39	27.6298	0.4975	0.5295	0.6924
35	525.12	4.9815	0.7909	0.2875	0.4466
36	549.4	35.7831	0.1993	0.6627	0.7971
37	556.34	12.2934	0.5221	0.7019	0.8249
38	575.1	16.8783	0.8337	0.7084	0.8293
39	589.69	49.2976	0.4907	0.5221	0.686
40	598.64	9.1459	2.3763	0.7223	0.8387
41	616.53	23.7222	1.4372	0.6721	0.8039
42	645.93	88.7004	2.0376	0.675	0.806
43	652.65	209.4184	2.695	0.6419	0.7819
44	687.24	16.757	2.2717	0.3309	0.4973
45	707.29	33.769	0.523	0.2544	0.4056
46	723.82	10.0294	12.2383	0.0082	0.0163
47	755.99	5.5182	0.4785	0.591	0.7429
48	775.56	23.5361	6.8154	0.0495	0.0944
49	885.85	3.9364	1.7215	0.1349	0.2377
50	1028.15	62.0524	0.5962	0.7044	0.8266
51	1123.16	21.7406	0.4681	0.1172	0.2098
52	1148.58	13.4303	0.1325	0.3188	0.4835
53	1159.54	131.1456	0.8303	0.2988	0.4601
54	1174.23	220.2934	0.192	0.5532	0.7123
55	1185.66	46.1836	2.0322	0.6115	0.7589
56	1195.99	20.2279	0.3333	0.718	0.8359
57	1201.04	47.3264	0.8427	0.6935	0.819
58	1211.95	81.7718	0.747	0.6992	0.823
59	1215.29	66.7758	2.0367	0.7338	0.8465
60	1225.69	74.9402	0.4892	0.3747	0.5451
61	1232.76	94.8169	1.0515	0.5926	0.7442
62	1237.17	401.4585	1.1673	0.7455	0.8542
63	1246.99	148.5616	1.1903	0.7388	0.8498
64	1268.51	355.0353	0.8374	0.75	0.8571
65	1276.12	239.5648	0.8575	0.5175	0.682
66	1296.32	31.1806	1.486	0.5928	0.7443
67	1314.94	18.9041	5.0261	0.7126	0.8322
68	1335.45	40.8968	8.7304	0.7083	0.8293
69	1379.93	73.7858	11.8772	0.75	0.8571
70	1415.4	47.7267	5.7625	0.7494	0.8567
71	1874.74	252.0703	7.5669	0.3387	0.506
72	3745.61	83.0961	144.3164	0.3026	0.4647

Table S4, DFT calculation data of vibration mode of PFHxS

Mode #	Frequency cm^{-1}	Infrared Intensity	Raman Activity	Depolar-P	Depolar-U
1	22.89	1.3498	0.0318	0.7082	0.8292
2	31.64	0.0963	0.0088	0.7408	0.8511
3	36.74	0.7404	0.0243	0.6681	0.801
4	45.67	0.3799	0.0161	0.7343	0.8468
5	55.08	0.0407	0.008	0.4439	0.6149
6	64.92	0.1923	0.0049	0.1419	0.2486
7	75.5	0.463	0.0263	0.3716	0.5419
8	126.28	0.3889	0.4024	0.2399	0.387
9	135.34	0.0413	1.914	0.2125	0.3505
10	178.08	11.1751	1.0075	0.7314	0.8449
11	184.23	0.5686	0.5889	0.4801	0.6488
12	200.21	3.0024	0.3377	0.5017	0.6682
13	202.47	5.3001	0.071	0.5837	0.7372
14	220.15	29.0024	2.1049	0.6654	0.7991
15	224.22	3.0631	0.3991	0.5642	0.7214
16	237.14	0.8557	0.1155	0.7495	0.8568
17	252.93	0.1246	0.1416	0.6364	0.7778
18	254.46	0.1814	0.1574	0.7293	0.8434
19	273.3	29.9193	1.9334	0.2227	0.3643
20	288.1	2.8918	3.3857	0.4285	0.5999
21	289.61	14.6004	2.708	0.7282	0.8428
22	298.7	1.1725	4.3476	0.6473	0.7859
23	316.01	0.1921	0.8795	0.2631	0.4166
24	334.3	1.7892	0.4242	0.4712	0.6406
25	348.34	0.3221	0.3347	0.6326	0.775
26	362.28	0.1138	0.1616	0.1199	0.2142
27	369.03	0.2903	0.7022	0.7307	0.8444
28	376.25	0.7906	1.9227	0.3941	0.5654
29	392.65	9.2077	0.2449	0.5644	0.7216
30	426.35	35.4062	1.8754	0.7452	0.854
31	458.5	12.1149	0.9173	0.6663	0.7997
32	484.3	12.5193	2.1427	0.6576	0.7934
33	520.86	48.1283	1.2665	0.6404	0.7808
34	531.65	6.7403	1.2137	0.6827	0.8114
35	545.9	103.0561	0.708	0.7029	0.8256
36	554.37	48.4715	0.569	0.749	0.8565
37	580.05	12.4703	1.8087	0.6883	0.8154
38	597.27	43.5037	1.6931	0.1739	0.2963
39	601.55	6.332	1.6633	0.7466	0.8549

40	634.9	43.2931	1.1125	0.2997	0.4612
41	677.38	46.9089	3.1725	0.0483	0.0921
42	696.5	76.4421	0.895	0.516	0.6807
43	722.95	9.3719	14.3982	0.0091	0.0181
44	756.03	9.1248	1.5264	0.1994	0.3325
45	806.41	160.3064	9.596	0.0328	0.0636
46	899.02	27.6481	8.0534	0.2305	0.3746
47	1063.41	63.5445	6.8334	0.3683	0.5383
48	1126.77	31.0532	6.9277	0.3422	0.5099
49	1133.76	35.2841	3.905	0.4217	0.5932
50	1150.73	47.7252	0.5335	0.1346	0.2372
51	1170.35	87.7806	3.1586	0.0813	0.1504
52	1186.97	224.9801	5.7714	0.1992	0.3323
53	1202	3.1393	0.3767	0.7091	0.8298
54	1203.62	103.352	1.3259	0.4274	0.5989
55	1218.27	18.5269	1.9664	0.4282	0.5997
56	1221.17	78.9644	1.6916	0.7357	0.8478
57	1235.65	92.3899	0.6947	0.7098	0.8303
58	1243.2	302.0517	1.76	0.6252	0.7694
59	1253.07	34.2267	4.5785	0.3561	0.5251
60	1269.08	457.4888	0.8928	0.7439	0.8531
61	1279.09	190.2848	0.8774	0.3566	0.5258
62	1306.88	27.4045	4.5581	0.6227	0.7675
63	1330.4	36.3554	5.353	0.7491	0.8566
64	1378.51	62.7797	9.4669	0.7192	0.8367
65	1405.05	220.5806	6.7287	0.7392	0.85
66	3752.47	139.9064	69.496	0.2757	0.4322

Table S5, DFT calculation data of vibration mode of PFOS

Mode #	Frequency cm^{-1}	Infrared Intensity	Raman Activity	Depolar-P	Depolar-U
1	18.54	1.8357	0.019	0.7178	0.8357
2	26.26	0.3237	0.0047	0.713	0.8324
3	34.1	0.6371	0.013	0.7472	0.8553
4	35.41	0.1608	0.0035	0.6605	0.7956
5	52.64	0.0916	0.0044	0.714	0.8332
6	64.91	0.4475	0.0049	0.6976	0.8219
7	68.18	0.325	0.0039	0.5594	0.7174
8	73.35	0.0211	0.0016	0.6611	0.796
9	82.17	0.4097	0.0071	0.467	0.6367
10	117.97	0.4777	0.1008	0.2199	0.3606
11	124.13	0.0381	1.2541	0.2154	0.3544

12	166.81	0.5835	0.1276	0.3433	0.5111
13	194.45	1.4024	0.1207	0.5436	0.7043
14	203.52	12.3008	0.4371	0.7461	0.8546
15	220.36	10.0224	0.0671	0.7247	0.8404
16	222.5	0.7042	0.0709	0.4287	0.6001
17	232.21	0.7867	0.1132	0.7085	0.8294
18	237.32	1.9515	0.067	0.7439	0.8532
19	254.48	0.2547	0.1292	0.6973	0.8217
20	264.31	20.5681	1.559	0.5897	0.7419
21	268.6	0.1993	0.1193	0.7201	0.8372
22	274.06	7.878	0.6171	0.5678	0.7243
23	287.02	0.1028	0.1137	0.3356	0.5026
24	288.37	0.791	0.1957	0.5769	0.7317
25	319.24	7.895	0.9153	0.3265	0.4923
26	324.4	2.4761	2.4495	0.5459	0.7062
27	334.79	4.3221	3.6077	0.6471	0.7858
28	339.71	38.6026	1.1888	0.4915	0.6591
29	347.22	25.8727	0.5876	0.651	0.7886
30	362.28	2.908	0.348	0.4418	0.6129
31	371.67	0.6831	0.5129	0.573	0.7286
32	389.1	0.4691	0.3687	0.2448	0.3933
33	401.22	0.037	0.1231	0.7051	0.8271
34	406.6	0.2219	0.1162	0.5238	0.6875
35	410.46	3.6288	0.2364	0.4719	0.6412
36	412.36	0.9639	1.1787	0.7271	0.842
37	419.07	0.4396	1.5785	0.2062	0.3419
38	458.08	7.7768	0.1901	0.6588	0.7943
39	490.67	48.5885	0.9094	0.7298	0.8438
40	517.88	22.2607	0.5512	0.7372	0.8487
41	553.51	18.6506	0.9779	0.7449	0.8538
42	575.07	86.0422	0.855	0.5387	0.7002
43	584.15	21.9251	0.8629	0.7497	0.857
44	603.83	167.3363	0.5724	0.7361	0.848
45	606.27	101.8255	0.2979	0.7465	0.8549
46	618.2	4.0858	1.3562	0.7399	0.8505
47	629.34	18.9484	0.5742	0.714	0.8331
48	649.92	2.5615	0.2538	0.7383	0.8494
49	663.08	51.3868	2.5567	0.7483	0.856
50	681.41	112.5602	0.8252	0.4719	0.6412
51	707.63	42.1129	0.5834	0.1943	0.3254
52	729.38	135.2282	0.5755	0.7491	0.8565
53	757.1	32.0697	2.0815	0.0493	0.094
54	777.58	7.9411	0.2815	0.4426	0.6136

55	799.23	0.762	13.822	0.0075	0.015
56	822.34	6.7957	2.0449	0.038	0.0733
57	905.65	7.7487	6.9989	0.0245	0.0478
58	968.73	209.9628	5.2562	0.0849	0.1565
59	1074.51	52.8896	1.0307	0.6843	0.8126
60	1201.47	24.1248	2.7665	0.2117	0.3494
61	1241.29	79.6484	2.8066	0.6544	0.7911
62	1254.97	47.9678	1.5686	0.2904	0.4501
63	1266.9	20.1522	3.6069	0.1989	0.3318
64	1281.01	159.8829	0.977	0.0998	0.1815
65	1302.43	358.9683	0.4113	0.13	0.2301
66	1339.77	217.6094	5.1968	0.2892	0.4487
67	1362.74	9.5421	0.1006	0.3421	0.5098
68	1363.4	10.9849	0.2783	0.6947	0.8198
69	1370.18	14.4613	0.6512	0.677	0.8074
70	1374.17	8.9413	0.809	0.6776	0.8078
71	1388.77	290.1155	1.5648	0.5571	0.7155
72	1394.1	25.0251	2.177	0.7254	0.8408
73	1401.92	185.942	1.7369	0.7386	0.8497
74	1405.63	467.9822	0.8966	0.7269	0.8419
75	1413.27	147.3863	1.1365	0.75	0.8571
76	1428.33	143.8329	0.6775	0.6781	0.8082
77	1438.01	478.9132	1.3902	0.7499	0.8571
78	1464.61	45.1097	2.2277	0.3976	0.569
79	1483.89	36.3061	3.1619	0.7194	0.8368
80	1503.08	11.868	8.8907	0.75	0.8571
81	1530.06	60.4548	9.5863	0.6884	0.8154
82	1556.74	326.0479	2.874	0.742	0.8519
83	1575.67	70.3743	23.2385	0.7193	0.8367
84	4092.31	255.3752	48.262	0.2789	0.4361

Table S6, DFT calculation data of vibration mode of PFHpA

Mode #	Frequency cm^{-1}	Infrared Intensity	Raman Activity	Depolar-P	Depolar-U
1	26.85	0.9144	0.4657	0.7445	0.8535
2	33.58	1.1042	0.2881	0.7497	0.8569
3	36	0.5219	0.438	0.7482	0.856
4	46.32	0.0739	0.0283	0.7395	0.8503
5	53.38	0.06	0.0248	0.7498	0.857
6	62.56	0.156	0.0054	0.7492	0.8566
7	78.26	0.1379	0.1369	0.7275	0.8423

8	128.99	0.4885	0.3058	0.6936	0.8191
9	144.6	0.0461	1.1082	0.2487	0.3983
10	184.2	0.3318	0.1427	0.1551	0.2686
11	197.68	4.962	0.0639	0.7323	0.8455
12	205.11	0.6056	0.0598	0.7436	0.8529
13	215.74	0.2867	0.0082	0.7201	0.8373
14	231.95	0.1676	0.0206	0.7391	0.85
15	247.58	0.34	0.0795	0.734	0.8466
16	250.32	0.104	0.0145	0.582	0.7357
17	258.03	1.0704	0.0817	0.4902	0.6579
18	288.63	5.284	0.9167	0.3558	0.5249
19	307.88	0.1029	1.868	0.7492	0.8566
20	312.78	0.0224	2.6319	0.7409	0.8512
21	341.2	0.2779	0.1232	0.2566	0.4084
22	357.04	1.3512	0.1022	0.666	0.7995
23	366.08	1.1176	0.6833	0.313	0.4768
24	367.67	0.4454	0.4224	0.7244	0.8402
25	373.88	1.7643	2.5299	0.2516	0.4021
26	377.2	0.3532	1.1912	0.7479	0.8558
27	429.27	0.7065	0.2073	0.6316	0.7742
28	488.52	1.3628	0.2476	0.7221	0.8386
29	511.82	40.0943	1.2333	0.5846	0.7379
30	525.39	5.7202	0.6737	0.324	0.4895
31	544.47	11.4978	0.3425	0.5363	0.6982
32	557.64	15.8469	0.2844	0.3337	0.5004
33	577.04	43.1454	0.686	0.7425	0.8522
34	592.51	11.3832	1.9058	0.7056	0.8274
35	608.65	12.1125	1.7341	0.6936	0.8191
36	629.8	23.7895	0.3479	0.5686	0.7249
37	649.08	242.2167	3.6336	0.6436	0.7832
38	678.35	46.3517	2.7256	0.4507	0.6214
39	708.58	17.4403	0.4731	0.159	0.2744
40	728.61	27.7801	12.5181	0.0179	0.0351
41	762.27	16.9449	1.5321	0.3375	0.5047
42	786.7	30.2939	3.8585	0.0438	0.0838
43	935.61	4.4775	1.6448	0.1409	0.247
44	1081.24	76.0951	0.4455	0.7289	0.8432
45	1145.25	3.0296	0.2544	0.1511	0.2625
46	1156.44	101.2246	0.4117	0.3847	0.5557
47	1172	189.5095	0.6126	0.3364	0.5034
48	1186.08	53.0335	2.292	0.6381	0.7791
49	1199.64	8.0391	0.3156	0.7464	0.8548
50	1203.6	192.8182	1.1449	0.6541	0.7909

51	1218.5	104.7236	1.3392	0.667	0.8003
52	1220.58	76.3726	1.4389	0.5683	0.7248
53	1228.76	178.3859	1.1396	0.7349	0.8472
54	1243.47	167.8519	0.7079	0.5316	0.6942
55	1256.77	70.5035	0.9399	0.5497	0.7094
56	1270.18	507.2744	0.7281	0.6461	0.785
57	1278.07	98.1802	1.2395	0.7317	0.8451
58	1309.88	47.6745	4.4874	0.6888	0.8157
59	1331.21	23.1192	6.1606	0.7309	0.8445
60	1376.15	85.5562	10.0699	0.7482	0.856
61	1415.61	39.0108	4.7632	0.7435	0.8529
62	1874.63	252.2551	7.5631	0.3354	0.5023
63	3745.74	82.5599	141.8376	0.3023	0.4643

Table S7: LC-ESI-MS/MS conditions and parameters for PFAS analytes

#	List of PFAS	CAS#	Abbreviation	Molecular Weight	Molecular Ion	Product Ion	Declustering potential (volts)	Entrance Potential (volts)	Collision Energy (volts)	Exit Potential (volts)	Retention Time (min)	Limit of Detection (ng/ml)	R^2 Value
1	Perfluoro-n-butanoic Acid	375-22-4	PFBuA	214.06	213	169	-32	-10	-13	-10	6.1	<0.007	0.9995
2	Perfluorohexanoic acid	307-24-4	PFHxA	314.06	313	269	-35	-10	-13	-12	7.4	<0.0015	0.9998
3	Perfluoroheptanoic acid	375-85-9	PFHpA	364.06	363	168.6	-40	-10	-24	-14	8.3	<0.002	0.9998
4	Perfluorooctanoic acid	335-67-1	PFOA	414	413	168.6	-40	-10	-24	-14	9.3	<0.002	0.9995
5	Perfluorononanoic acid	375-95-1	PFNA	464.07	463	419	-40	-10	-15	-10	10.4	<0.001	0.9997
6	Perfluorooctane-sulfonate	1763-23-1	PFOS	500	499	98.8	-80	-10	-60	-10	10.2	<0.007	1.000
7	Perfluorodecanoic acid	335-76-2	PFDA	514.08	513	469	-45	-10	-15	-10	11.5	<0.0015	0.9994
8	Perfluoroundecanoic acid	2058-94-8	PFUdA	564.08	563	519	-40	-10	-15	-10	12.4	<0.005	0.9999
9	Perfluorododecanoic acid	307-55-1	PFDoA	614.08	613	569	-45	-10	-17	-13	13.3	<0.002	0.9996

10	Undecafluoro-2-methyl-3oxahexanoic Acid	13252-13-6	GenX	330.05	329.2	285	-25	-10	-8	-7	7.7	<0.03	0.9998
11	N-methylperfluro-octanesulfonamidoacetic acid	2355-31-9	Me-PFOSAA	571.2	570	419	-65	-10	-28	-9	12.1	<0.05	0.9999
12	N-ethylperfluro-octanesulfonamidoacetic acid	2991-50-6	Et-PFOSAA	585.2	584	419	-70	-10	-29	-21	12.6	<0.04	1.000

Table S8: Total fluorine of commercial AFFF acquired by LC-MS/MS and Combustion Ion Chromatography (CIC)

Foam name	Total F by LC-MS/MS (ng/mg)	Total F by CIC (mcg/mL)	Ratio MS/CIC	Foam density (g/mL)
AFFF- FC-201F	32563	62231	0.48	1.08

* Ratio takes into account foam density, 32563/ (62231*1.08) for mcg/g
